# Heterogeneity and Fate Regulation of Fibrocartilage Stem Cells

**DOI:** 10.3390/biology15212017

**Published:** 2026-11-06

**Authors:** Shuo Chen, Yang He

**Affiliations:** Department of Oral and Maxillofacial Surgery, Peking University School and Hospital of Stomatology & National Center for Stomatology & National Clinical Research Center for Oral Diseases & National Engineering Research Center of Oral Biomaterials and Digital Medical Devices, Beijing 100081, China; chens@bjmu.edu.cn

**Keywords:** temporomandibular joint, fibrocartilage stem cells, osteoarthritis, cellular heterogeneity, cell fate determination

## Abstract

The temporomandibular joint (TMJ) is one of the most complex and most frequently used synovial joints. The condylar surface is covered by a distinctive type of fibrocartilage that possesses a remarkable capacity for adaptive remodeling. In recent years, with rapid advances of single-cell transcriptomics and in vivo lineage-tracing technologies, fibrocartilage stem cells (FCSCs) residing in the superficial layer of the mandibular condyle have been demonstrated to play a decisive role in TMJ development, homeostasis, and the pathological progression of temporomandibular joint osteoarthritis (TMJOA). However, FCSCs do not constitute a single homogeneous population but rather exhibit substantial lineage heterogeneity. Under the spatiotemporal orchestration of distinct mechanical microenvironments and molecular signaling networks, different subpopulations of stem/progenitor cells adopt divergent fate trajectories toward chondrogenic or osteogenic differentiation. This review focuses on the cellular heterogeneity of FCSCs and the regulatory mechanisms governing their fate determination, summarizing recent advances in the field and discussing the translational potential of these findings for TMJ regenerative medicine and targeted therapeutic strategies.

## Simple Summary

The temporomandibular joint connects the lower jaw to the skull. It is one of the most complex and heavily used joints in the body. The joint surface is covered by a special flexible tissue called fibrocartilage, which helps the joint absorb pressure and adapt to daily wear and tear. In recent years, new tools have allowed scientists to study individual cells one at a time. These studies show that a group of stem cells lives in the outer layer of this flexible tissue. These stem cells are important for joint growth and normal upkeep, and they are also involved in the development of jaw joint arthritis. However, these stem cells are not all the same. They differ in their abilities, and depending on the mechanical forces and chemical signals around them, they can turn into either cartilage-making cells or bone-making cells. This review summarizes what we know about these differences and how the fate of these stem cells is controlled. It also discusses how this knowledge could help doctors regenerate jaw joint cartilage and develop better treatments for arthritis.

## 1. Introduction

The mandibular condylar cartilage of the temporomandibular joint (TMJ) is a secondary cartilage, which differs significantly from the primary hyaline cartilage of long bones in terms of histological origin, biomechanical responsiveness, and disease progression [1,2]. Unlike hyaline cartilage, fibrocartilage consists of both fibrous and cartilaginous components, which confer tensile and compressive strength [3]. Typically, condylar fibrocartilage is divided into four layers including the superficial zone (SZ), polymorphic zone (PZ), flattened chondrocyte zone (CZ), and hypertrophic chondrocyte zone (HZ) [4]. Within the SZ of the condylar cartilage resides a population of stem cells endowed with self-renewal capacity and multipotent differentiation potential—the fibrocartilage stem cells (FCSCs) [5]. Since Embree et al. first identified FCSCs and demonstrated their essential role in cartilage repair [5], FCSCs have rapidly emerged as a central focus in studies of TMJ developmental biology and disease mechanisms [6,7].

TMJ osteoarthritis (TMJOA) is a common degenerative disorder of the craniofacial region [8,9,10,11]. Beyond the joint itself, temporomandibular disorders are increasingly recognized as having consequences for general health, including altered postural stability [12]. Traditionally, TMJOA pathogenesis has been attributed predominantly to cartilage matrix degradation and inflammatory cytokine-mediated destruction [13]. However, recent research paradigms have undergone a profound shift: the essence of TMJOA lies in the exhaustion of the FCSC reservoir induced by specific pathological mechanical or inflammatory microenvironments, coupled with divergent transduction of their progeny—namely, a loss of chondrogenic differentiation capacity and aberrant hypertrophy with osteogenic commitment [6,14].

In this context, a comprehensive dissection of FCSC lineage heterogeneity, along with elucidation of the regulatory mechanisms governing their fate determination under distinct physiological and pathological loading conditions, may ultimately reshape our understanding of TMJ development and growth [6,7]. Exogenous cell-based therapies tested in large-joint osteoarthritis have yielded results that remain controversial; for example, their long-term efficacy has not yet been established. This further underscores the rationale for endogenous strategies that harness the joint’s own regenerative reservoir [15].

Thereby, this review aims to deepen the understanding of FCSC lineages by synthesizing current evidence on their lineage heterogeneity, the signaling networks governing their fate, and their niche interactions in homeostasis and TMJOA, with particular emphasis on cross-study comparison, critical appraisal of the available evidence, and translational implications.

## 2. Methods

### 2.1. Search Strategy

This narrative review was conducted and reported in accordance with established reporting principles for clinical and basic research reviews [16]. We performed literature search of PubMed, Web of Science, and Embase for articles published between January 2016 and August 2026, using MeSH terms and free-text terms with the following search string: (“temporomandibular joint” OR “mandibular condyle” OR condylar) AND (“fibrocartilage stem cell” OR FCSC OR progenitor OR “stem cell”) AND (heterogeneity OR lineage OR fate OR niche OR signaling OR “single-cell” OR mechanotransduction). The final search was conducted on 31 August 2026.

### 2.2. Inclusion and Exclusion Criteria

Eligible publications were original basic research articles and landmark reviews focusing on FCSCs and TMJ condylar cartilage biology. Conference abstracts, editorials, preprints, in-press articles, and non-English articles were excluded.

### 2.3. Data Collection and Extraction

After removal of duplicates, the search yielded 110 records. Reference lists of key original articles and reviews were manually screened to capture additional relevant publications, including articles published prior to 2016. Studies were selected based on their relevance, methodological rigor, and contribution to the thematic synthesis.

## 3. Cellular Atlas Reconstruction: Heterogeneity of FCSCs

Stem cells possess the capacity for self-renewal and multilineage differentiation. Upon lineage commitment, they give rise to progenitor cells with restricted differentiation potential and limited self-renewal, which expand and differentiate to generate lineage output. Stem/progenitor cells is an umbrella term referring to a mixed population containing stem cells and their lineage-committed progeny. Sometimes, the term “stem/progenitor cells” is used as a collective nomenclature when stem cells and progenitor cells cannot be fully resolved into distinct populations. In this review, we follow the original wording of cited references to preserve objectivity.

5-ethynyl-2′-deoxyuridine (EdU) label retention has been extensively employed to identify slow-cycling stem cell populations. In previous studies, label-retaining cells (LRCs) were shown to reside within the SZ, suggesting that this region may house an endogenous FCSCs population [5]. A lineage-tracing study showed that undifferentiated alpha-smooth muscle actin (α-SMA)+ cells give rise to mature collagen type II alpha 1 chain (Col2a1)+ chondrocyte progeny in condylar cartilage. Remarkably, a single FCSC can spontaneously generate cartilage, form bone, and organize a hematopoietic microenvironment upon transplantation, without the need for exogenous osteogenic factors [5]. Collectively, Embree et al. were first to demonstrate that the fibrous SZ tissue of the TMJ condyle constitutes a niche housing FCSCs. Bi et al. subsequently identified endogenous stem cells in the SZ of the human TMJ condyle and termed them human fibrocartilage stem cells (hFCSCs) [17]. These findings highlight the potential of harnessing endogenous FCSCs as a regenerative strategy that bypasses cell transplantation, which could offer therapeutic benefits to patients with fibrocartilage injury and disease [5,18].

Earlier studies tended to view SZ cells of the condyle as a homogeneous pool of FCSCs. However, over the past ten years, single-cell RNA sequencing (scRNA-seq) and rigorous Cre-loxP lineage tracing techniques have thoroughly overturned this notion of homogeneity, revealing a heterogeneous landscape of stem/progenitor cells characterized by interwoven multi-lineage contributions and functionally distinct subsets [5,19,20,21,22,23,24].

### 3.1. Scx+ Lineage: A Subset of Tendon-Derived Chondrocytes

Scleraxis (Scx) has traditionally been regarded as a specific marker for tendons and ligaments [25,26]. However, lineage-tracing studies have revealed that, within the complex anatomical architecture of the TMJ, Scx+ tendon/ligament-derived cells can undergo directed chondrogenic differentiation and contribute directly to the formation of TMJ chondrocytes. This finding challenges the conventional tissue boundaries and offers a novel perspective on the integrated development of the disc–condyle complex [19].

In the *Scx-Cre^ERT2^*; *tdTomato* lineage-tracing mouse model, Ma et al. found that Scx+ cells, traditionally considered tendon/ligament progenitors, directly contribute to the formation of the TMJ disc and a distinct subset of condylar chondrocytes. These Scx+ cells originate from the fibrous layer, first transitioning into dendritic prechondroblasts (SRY-box transcription factor 9 [Sox9]-/collagen type I [Col I]+) and subsequently differentiating into mature chondrocytes (Sox9+/aggrecan [Acan]+, Col I −). Importantly, these Scx-derived chondrocytes do not undergo further differentiation into bone cells, unlike the non Scx-chondrocyte population, which gives rise to the majority of subchondral osteoblasts and osteocytes. This finding identifies Scx+ lineage chondrocytes as a “permanent” chondrocyte pool that maintains cartilage mass without contributing to endochondral ossification [19].

Furthermore, the functional significance of this lineage was further explored in dentin matrix protein 1 (Dmp1)-null mice, a model of hypophosphatemic rickets. In these animals, the number of Scx-derived chondrocytes increased sharply (approximately 2.5-fold, *n* = 4), along with enhanced aggrecan expression and expanded hypertrophic zones, whereas no Scx-derived bone cells were observed. This suggests that the Scx-lineage chondrocyte subset is highly sensitive to phosphorus levels and is responsible for the disproportionate cartilage expansion seen in hypophosphatemia, without contributing to bone formation [19].

Collectively, these findings challenge the traditional view that TMJ chondrocytes arise solely from the perichondrium [1,2]. They establish that Scx-lineage cells play dual roles in TMJ development: forming the articular disc and generating a unique chondrocyte population that remains permanently cartilaginous throughout the postnatal period examined (up to 8 months of age), maintaining cartilage mass without osteogenic differentiation, and expands markedly in response to hypophosphatemia in Dmp1-null mice [19]. This work provides new insights into TMJ biology, highlights the heterogeneity of chondrocyte lineages, and suggests that tendon-derived cells may have broader developmental functions beyond mechanical support. The study also has potential implications for understanding TMJ disorders and hypophosphatemic bone diseases.

### 3.2. Gli1+ Lineage: A Key Population in Cartilage Homeostasis and Bone Remodeling

Gli1 serves as a readout of the Hedgehog (Hh) signaling pathway and is widely used to identify Hh-responsive cells. In various tissues, including the dental pulp, periodontal ligament, and cranial sutures, Gli1 has also been used to label stem cells [27,28,29]. Interestingly, recent studies demonstrated that Gli1+ cells are distributed in both the superficial zone of the fibrocartilage and the subchondral bone [20,30,31,32,33,34].

Wang et al. initially identified Gli1 as a potential stem cell marker using single-cell RNA sequencing and pseudotime trajectory analysis, which positioned Gli1+ cells at the very beginning of the condylar cartilage differentiation process. Gli1+ cells are strictly localized within the superficial zone—the recognized niche for FCSCs—and are spatially separated from Sox9+ chondroprogenitors. Furthermore, LRCs assays indicated that roughly 19.95% ± 4.44% of Gli1+ cells were quiescent LRCs, and Gli1 expression was detected in 25.33% ± 7.85% of all LRCs (*n* = 3). This finding further suggests the existence of heterogeneity within the FCSC population [20]. In vivo, continuous lineage tracing revealed that Gli1+ cells not only self-renew to maintain the stem cell pool but also actively migrate and differentiate into mature and hypertrophic chondrocytes. Finally, targeted genetic ablation of these cells (*Gli1-Cre^ERT2^*; *DTA*) resulted in a depleted SZ and significantly reduced cartilage thickness.

In TMJOA, Gli1+ FCSCs initially undergo compensatory activation, with enhanced proliferation and chondrogenic differentiation toward Sox9+ and collagen type X (Col X)+ chondrocytes. However, they subsequently become exhausted and lose their regenerative capacity, leading to cartilage degradation, impaired subchondral bone remodeling, and mandibular growth retardation [20,32]. Early disc repositioning reverses this pathological fate by restoring Gli1+ FCSC proliferation and chondrogenic potential, thereby re-establishing cartilage homeostasis and promoting condylar growth in growing mice [32]. Collectively, these results proved that Gli1 marks an indispensable FCSC subpopulation required for condylar cartilage development and tissue homeostasis.

Interestingly, Gli1+ cells residing at the chondro-osseous junction act as osteogenic progenitors. They give rise to nearly all osteoblasts in the subchondral bone, making them essential for postnatal bone formation, tissue homeostasis, and adaptive remodeling in the mandibular condyle. Upon condylar fracture, the Gli1+ skeletal progenitor pool is activated. These cells migrate toward the fracture callus and differentiate into both osteoblasts and chondrocytes to facilitate bone regeneration and healing [30]. In the context of osteoarthritis induced by aberrant mechanical loading, Gli1+ osteogenic progenitors undergo excessive expansion and increased, unevenly distributed osteoblastic differentiation. This overactivity disrupts subchondral bone homeostasis, leading to abnormal bone remodeling (such as altered trabecular architecture and enlarged marrow cavities), which consequently impairs the overlying cartilage and exacerbates osteoarthritis (OA) [31,34,35].

### 3.3. Ctsk+ Lineage: A Mechanosensitive Subpopulation in Cartilage Homeostasis

Cathepsin K (Ctsk) is a collagenolytic protease traditionally linked to bone resorption and osteoarthritic cartilage degradation. Beyond its role in matrix turnover, Ctsk has been widely used as a marker to identify progenitor populations in the periosteum, jawbone, tendon, craniofacial tissues, and nucleus pulposus [36,37,38,39,40].

Ctsk-driven lineage tracing showed that labeled cells were initially present only in the SZ of 3-day-old condylar cartilage. By the age of two weeks, these cells had descended into the deeper cartilage layers and had undergone differentiation into Col2+ mature chondrocytes. This time-course data thus supports the notion that Ctsk can serve as a faithful tracer for a distinct FCSCs subpopulation in vivo [23].

Ctsk+ FCSCs are sensitive to biomechanical stimuli, such as tensile stress generated by mandibular advancement. This mechanical loading causes an initial, rapid burst of chondrogenic differentiation, leading to a transient shrinkage of the FCSC pool, which is later replenished as the cells’ proliferation rate accelerates. However, during TMJOA, the proliferation and differentiation capacities of FCSCs are reduced [41]. In conclusion, Ctsk+ FCSCs are mechanosensitive and play an indispensable role in maintaining cartilage homeostasis. Because Ctsk is also highly expressed by osteoclasts, lineage data obtained with Ctsk-Cre drivers should be interpreted with the caveat that recombination in the osteoclast lineage may contribute to the observed phenotypes.

### 3.4. FSP1+ Lineage: Essential for TMJ Homeostasis and Growth

Fibroblast-specific protein 1 (FSP1), a calcium-binding member of the S100 family, is implicated in fibrosis, cirrhosis, pulmonary disease, cardiac hypertrophy, neuronal injury, and cancer [42,43]. It is expressed in diverse cell types, including fibroblasts, endothelial and smooth muscle cells, immune cells, bone cells, and chondrocytes of elastic and fibrocartilage [42,44,45]. Functionally, FSP1 regulates cell motility, viability, differentiation, and contractility via both intracellular mechanisms and paracrine signaling [43,44,46].

FSP1 is expressed in the superficial fibroblast zone of the TMJ condyle. These cells have been identified as neural crest-derived condyle fibroblasts [21]. FSP1 is not expressed during embryogenesis (e.g., at E17.5) but begins to appear in a few cells in the uppermost layers of the condyle at postnatal day 1 (P1). The number of FSP1-expressing cells increases significantly as the condyle matures, with the expression domain around the time of tooth eruption and weaning [21]. A subset of FSP1+ cells overlapped with Gli1+ and Scx+ cells, suggesting that these cells had stem cell-like properties. Only a small subpopulation of FSP1+ cells were LRCs (demonstrated by their ability to retain 5-bromo-2′-deoxyuridine [BrdU] labels long-term). Notably, all BrdU-labeled cells within the superficial layers of the condyle expressed FSP1, indicating that FSP1 marks a previously unrecognized stem/progenitor cell pool in the TMJ. Lineage tracing showed that FSP1+ stem/progenitor cells migrate into all underlying layers of the condyle and differentiate over time, eventually reaching the hypertrophic zone and ossified regions [21]. FSP1+ cells also contributed to subchondral bone formation, in contrast to the Scx+ lineage, which differentiates only into prechondroblasts and chondrocytes without further differentiating into bone cells [19].

Selective ablation of FSP1-expressing cells in *FSP1-Cre*; *DTA* mice caused progressive loss of the condylar superficial fibrocartilage, accompanied by increased apoptosis and reduced numbers of FSP1+ and Sox9+ cells. By 16 weeks of age, mutant condyles exhibited pronounced osteoarthritic change, including disorganized collagen II distribution, enhanced extracellular matrix remodeling, and grade 6 osteoarthritis according to Osteoarthritis Research Society International (OARSI) histopathological scoring of safranin O–stained sections (*n* = 3, blindly scored by observers). Micro-computed tomography further revealed posterior condylar enlargement, a concave articular surface with ectopic mineralization, and significantly shorter dentary (voxel size, scanner manufacturer and model were not reported in the original study) [21]. Collectively, these findings demonstrate that FSP1+ stem/progenitor cells are essential for maintaining TMJ cartilage homeostasis and normal mandibular growth. Of note, FSP1-Cre also recombines in hematopoietic and immune cells, and this recombination may contribute to the severity of the ablation phenotype; this alternative interpretation should be considered when attributing the phenotype exclusively to FCSC loss.

### 3.5. Lgr5+ Lineage: A Niche Cell Population Maintaining Cartilage Homeostasis

Leucine-rich repeat-containing G protein-coupled receptor 5 (Lgr5) was initially identified as a core marker for adult stem cells in the small intestine; however, due to the presence of a quiescent reserve stem cell population, these active Lgr5+ cells are not strictly indispensable for tissue regeneration [47,48]. Subsequently, research on this classic marker was extended to the oral domain, demonstrating that Lgr5 also specifically identifies a distinct population of neural crest-derived oral stromal stem cells possessing self-renewal and multipotent differentiation capacities [49].

Ruscitto et al. identify a subpopulation of Lgr5-expressing cells, which localized in the SZ stem cell niche of TMJ condyle. Lgr5+ cells are enriched in the SZ of young mandibular condylar cartilage, with reduced numbers during ageing and osteoarthritis. Lineage tracing shows that Lgr5+ cells supply progeny to the synovial joint meniscus/disc, the perichondrium’s superficial zone, the periosteum, and bone. However, they do not differentiate into chondroprogenitor cells (CPCs) or chondrocytes.

During development, Lgr5+ cells are essential for mandibular condyle morphogenesis; their ablation disrupts the condylar blastema, reduces the CPCs pool, and triggers premature chondrocyte hypertrophy and osteoblast-like differentiation. In adult homeostasis, they provide a local Wnt-inhibitory microenvironment that keeps adjacent CPCs in a Wnt-off state and preserves chondrocyte phenotypic stability. Loss of Lgr5+ cells—whether by genetic ablation, aging, or OA—leads to chondrocyte phenotypic instability, marked by ectopic runt-related transcription factor 2 (Runx2) and osteocalcin (OCN) expression, matrix degradation, and cartilage breakdown. Upon cartilage injury, Lgr5+ cells self-renew (increased EdU uptake and Lgr5 expression) but do not contribute directly to repair as progenitor cells; instead, they amplify their niche-supporting role to counter Wnt hyperactivity in damaged chondrocytes [22]. Mechanically, these Lgr5+ cells support a Wnt inhibitory niche, most probably through producing the Wnt antagonist Dickkopf 3 (DKK3), which cues adjacent CPCs to sustain low β-catenin levels and an undifferentiated state. Therefore, these Lgr5+ population was defined as secretory niche cells, providing a supportive role to CPCs and chondrocytes [22].

Most recently, single-cell transcriptomic profiling identified a discrete cellular retinoic acid-binding protein 1 (Crabp1) + population in the inner perichondrial/polymorphic zone of embryonic and neonatal condyles [24]. These Crabp1+ cells are spatially and transcriptionally distinct from the Lgr5+ superficial-zone niche cells and Sox9+ chondrogenic cells described above. Pseudotime and RNA velocity analyses place these cells at an early, undifferentiated node of a putative chondrogenic trajectory that partially overlaps Acta2+ mesenchymal populations. However, this assignment rests entirely on transcriptomic inference rather than on in vivo lineage tracing [24]. Crabp1+ cells should therefore be regarded as a transcriptomically defined candidate progenitor population, whose relationship to the lineage-traced FCSC compartments above remains to be validated by genetic lineage tracing. Table 1 summarizes the lineage-tracing evidence for these populations.

### 3.6. Comparative Overview of FCSC Populations

Although identified with different markers and experimental designs, these populations share three unifying features: preferential localization to the superficial fibrous region of the condylar cartilage, slow-cycling or label-retaining behavior, and the capacity to sustain fibrocartilage. They differ in three dimensions. First, anatomical localization: Scx+, Ctsk+, FSP1+, and superficial-zone Gli1+ cells reside in the SZ, whereas a spatially distinct Gli1+ population occupies the chondro-osseous junction. Second, lineage output: Scx+, Ctsk+, and superficial-zone Gli1+ cells are predominantly chondrogenic, whereas chondro-osseous-junction Gli1+ cells are osteogenic; Scx+ progeny give rise to chondrocytes but not bone cells, whereas FSP1+ progeny contribute to both cartilage and subchondral bone, and Lgr5+ cells act mainly as secretory niche cells. Third, evidence strength: most populations rest on in vivo genetic lineage tracing, whereas Crabp1+ cells are supported only by transcriptomic and spatial data and are putative.

**Figure 1:**
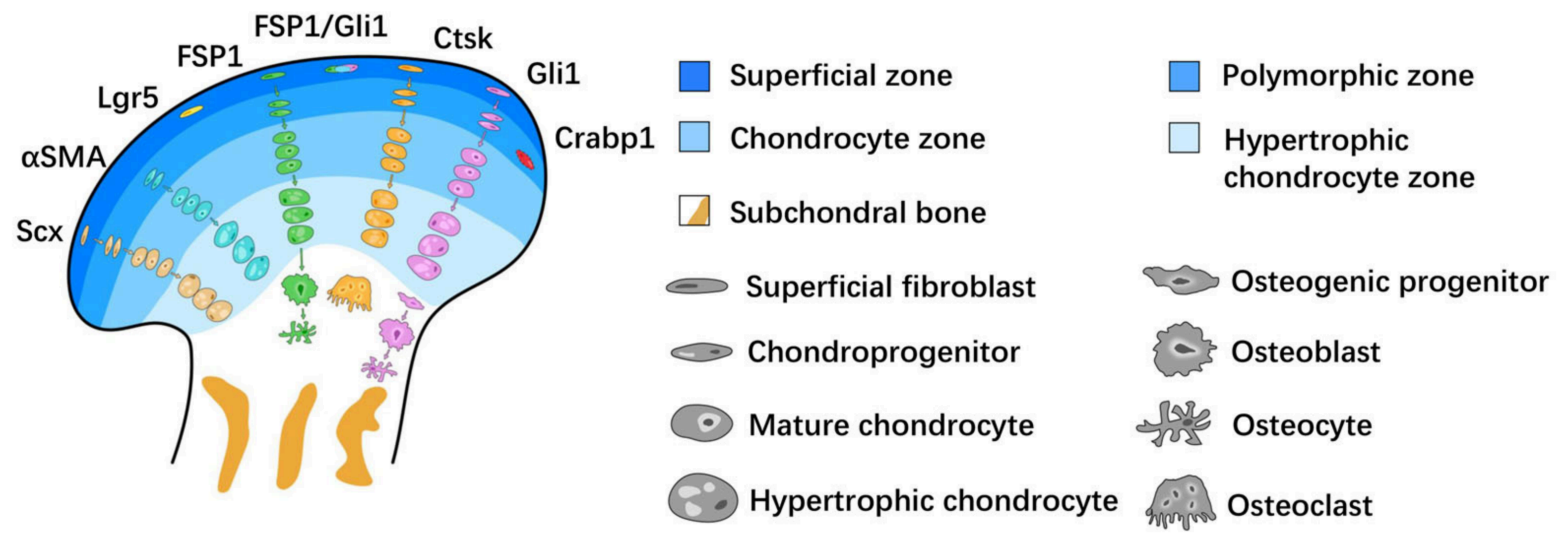
Lineage tracing schematic of Scx+ (pale yellow), α-SMA+ (cyan), Lgr5+ (yellow), FSP1+ (green), Ctsk+ (orange), Gli1+ (purple), and Crabp1+ (red) cells, and their contributions for cartilage or subchondral bone. Arrows indicate the direction of sequential differentiation from stem/progenitor cells to mature chondrocytes/osteocytes. The outline of Crabp1^+^ cell is marked with a dashed line, indicating this is a putative cell population, without lineage tracing.

Several questions about the overlap between these populations remain unresolved. The two Gli1+ compartments are anatomically and functionally distinct subpopulations that share an Hh-responsive marker but differ in lineage output. Whether FSP1+ cells constitute a subset of the Gli1+ compartment or merely overlap with it has not been tested by dual-lineage or combinatorial reporters. We propose that FSP1+ and Scx+ cells are separate: progeny of the former differentiating into hypertrophic chondrocytes and then undergoing osteogenesis, whereas those of the latter arrest at the hypertrophic stage. Lgr5+ secretory cells maintain the FCSC compartment as niche-supporting cells rather than as FCSCs themselves. These Lgr5+ cells are adjacent to yet mutually exclusive from the other compartments, since they lack chondrogenic differentiation. The putative Crabp1+ population awaits lineage-tracing validation. We summarized the lineage tracing findings and their overlapping patterns in Figure 1.

## 4. Regulatory Networks Shaping Stem Cell Fate

**Figure 2:**
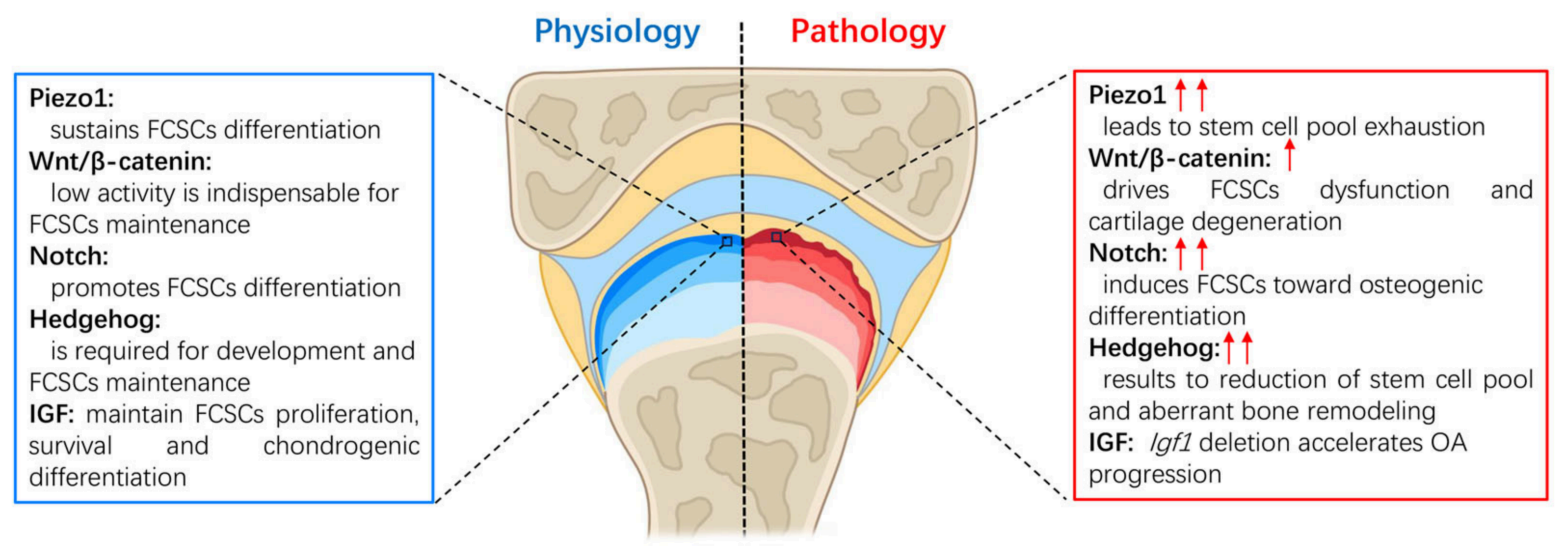
Signaling pathways governing fibrocartilage stem cell (FCSC) fate in the temporomandibular joint (TMJ) condyle under physiological and pathological conditions. Red arrows indicate upregulation of the signaling.

FCSC fate determination is controlled by an intricate regulatory network integrating the Wnt, Hh, and Notch pathways, as well as mechanotransduction signals. Dysregulation of these networks under pathological mechanical loading induces FCSC exhaustion and aberrant osteogenic differentiation, which contributes to the progression of TMJOA (Figure 2).

### 4.1. Piezo1 Mechanosensitive Ion Channel

Piezo-type mechanosensitive ion channel component 1 (Piezo1), a mechanically activated cation channel, serves as a critical mechanotransducer in cartilage and bone formation as well as homeostasis [50,51,52,53,54,55]. In the TMJ, Piezo1 plays a context-dependent role: essential for physiological chondrogenesis, but detrimental when aberrantly overactivated under pathological mechanical loading [33].

scRNA sequencing of human condylar tissue (condylar fragments from a single donor, a 16-year-old male who underwent surgery for a condylar fracture) identified PIEZO1 as the most abundantly expressed mechanosensitive ion channel in both cartilage and bone, surpassing transient receptor potential (TRP) family members [33]. Because this human atlas rests on a single donor, these expression data should be regarded as hypothesis-generating pending replication in additional donors. In an animal model, *Piezo1-tdTomato* reporter mice reveal Piezo1 expression in the condyle cartilage from P3.5 to 3 months of age, with expression emerging at 2 weeks, peaking at 6–8 weeks, and declining thereafter—a pattern coinciding with the onset of masticatory loading [33]. Conditional knockout of *Piezo1* in Gli1+ FCSCs (*Gli1-Cre^ERT2^*; *Piezo1^fl^*^/*fl*^) at postnatal week 1 results in significant cartilage thinning, reduced proteoglycan content, and decreased Sox9+ chondrocytes and Col X+ hypertrophic chondrocytes. Importantly, Gli1+ cells accumulated in the SZ/PZ without differentiating, showing increased Ki67+ proliferation but impaired chondrogenic capacity. This suggests that Piezo1 deletion blocks FCSC differentiation rather than proliferation, leading to progenitor accumulation and defective endochondral ossification [33].

Similarly, Gong et al. demonstrated that tensile stress induced by mandibular advancement (i.e., in 5-week-old male *Ctsk-Cre*; *tdTomato* mice, the lower incisors were trimmed by 1 mm every 3 days over a two-week period) accelerated FCSC chondrogenesis, as shown by Ctsk-based lineage tracing: the percentage of Ctsk+/Col2+ cells in the posterior condylar cartilage rose from 3.91% ± 1.08% (Sham) to 15.28% ± 2.82% (MA) at 1 week and remained elevated at 2 weeks (4.44% ± 1.96% vs. 11.1% ± 2.60%), whereas the percentage of Ctsk+/EdU+ cells increased only at 2 weeks (from 0.40% ± 0.13% to 3.05% ± 0.99%), indicating that tensile stress first drives chondrogenic differentiation of Ctsk+ FCSCs and subsequently their proliferation. Mechanistically, tensile stress upregulates chondrogenic markers (proteoglycan 4 [*Prg4*], *Col2a1*, and *Sox9*), and protein kinase C alpha (*Prkca*). Piezo1 inhibition with GsMTx4 or Ca^2+^ chelation with BAPTA abolished stretch-induced Ca^2+^ influx (10% equibiaxial dynamic strain, sine-wave pattern, 0.5 Hz, applied for 24 or 48 h at 37 °C on BioFlex 6-well plates in a Flexcell FX-6000 T system; Flexcell, Burlington, NC, USA) and reduced chondrogenic protein levels. Crucially, siRNA-mediated *Prkca* knockdown blocks tensile stress-induced *Col1*, *Col2*, and *Sox9* upregulation in vitro and reduces cartilage thickness and Col2/Sox9 expression in vivo, confirming the Piezo1–Ca^2+^–Prkca pathway as the core mechanotransduction axis [41].

In osteoarthritis, Piezo1 becomes a pathological driver. In the unilateral partial discectomy (UPD) mouse model, aberrant mechanical loading activates Gli1+ FCSCs, leading to their accelerated differentiation into hypertrophic chondrocytes and subsequent stem cell pool depletion at 4 weeks post-surgery. However, *Piezo1* deletion in Gli1+ FCSCs maintained the stem cell pool in the SZ and preserved cartilage thickness and matrix at 4 weeks after UPD [33]. In a unilateral anterior crossbite (UAC) model of TMJOA—established in 6-week-old female Sprague–Dawley rats by bonding metal tubes to the left maxillary and mandibular incisors, with condylar processes harvested at 2, 4 and 8 weeks after surgery; the magnitude of the resulting occlusal displacement was not reported—Piezo1 drives extracellular matrix degradation by promoting Yes-associated protein (YAP) nuclear translocation and upregulating the expression of matrix metalloproteinase 13 (MMP13) and a disintegrin and metalloproteinase with thrombospondin motifs 5 (ADAMTS5) [56]. In vitro, the same group loaded ATDC5 cells with fluid flow shear stress at 24 dyn/cm^2^ (2.4 Pa) using a Flexcell device, which reproduced the Piezo1–YAP–MMP13/ADAMTS5 response.

In addition, Zhou et al. demonstrated that mechanical loading activates Piezo1 in mesenchymal stem cells (MSCs) from subchondral bone, which triggers Ca^2+^ influx and subsequently upregulates signal transducer and activator of transcription 1 (STAT1)-mediated glycolysis via hexokinase 2 (HK2), leading to increased secretion of macrophage migration inhibitory factor (MIF). MIF in turn promotes T cell recruitment and T helper 17 (Th17) polarization, thereby driving subchondral bone erosion and cartilage degeneration in TMJOA. Conditional deletion of Piezo1 in MSCs (*Wnt1cre*; *Piezo1^fl^*^/*fl*^ mice) or inhibition of glycolysis effectively attenuates these pathological changes [57].

Thus, we propose a rheostat model as a working hypothesis: low-to-moderate Piezo1 activity sustains FCSC differentiation and cartilage growth, while excessive activity drives pathological stem cell exhaustion (Figure 2). This duality positions Piezo1 as a promising therapeutic target: transient activation may enhance cartilage remodeling, whereas pharmacological inhibition could protect against TMJOA progression by preserving the FCSC niche.

### 4.2. Wnt/β-Catenin Signaling

Wnt/β-catenin signaling plays a central, context-dependent role in regulating FCSCs within the TMJ condyle, governing their self-renewal, differentiation, and niche integrity across development, adult homeostasis, and osteoarthritis.

During development, Wnt activity is tightly spatiotemporally controlled. In the embryonic and early postnatal condyle, canonical Wnt activity is spatially partitioned rather than simply low or high: it is prominent in the outermost superficial, perichondrium-like layer (Axin2+ cells) as well as in maturing and hypertrophic chondrocytes, whereas the intervening progenitor layers—where FCSCs reside—are maintained in a Wnt-inactive state [5,20,21,22]. This niche is critical for proper condylar morphogenesis, as ablation of Lgr5+ cells during early development disrupts the condylar blastema and depletes Wnt-inactive progenitors. Notably, Wnt/β-catenin signaling in maturing and hypertrophic chondrocytes is indispensable for chondrogenic differentiation, since β-catenin deletion in Gli1+ cells impairs their differentiation into hypertrophic chondrocytes, leading to reduced cartilage thickness and disorganized zones [20].

In adults, sustained low Wnt activity is indispensable for FCSC maintenance and chondrocyte identity (Figure 2). The SZ niche, marked by Lgr5+ and later FSP1+ cells, continues to provide Wnt-inhibitory cues. FSP1 expression increases postnatally, coinciding with a progressive decline in the number of axis inhibition protein 2 (Axin2)+ Wnt-active cells in the superficial layers [21]. Constitutive activation of Wnt/β-catenin signaling in FSP1+ cells downregulates FSP1, depletes the progenitor pool, and disrupts cartilage architecture, demonstrating that Wnt suppression is required for FCSC niche integrity [21].

In osteoarthritis, Wnt signaling becomes pathologically overactivated, driving FCSC dysfunction and cartilage degeneration (Figure 2). In rabbit, rat and mouse TMJOA models (disc perforation or partial discectomy), ectopic β-catenin signaling expands into the SZ, leading to premature FCSC differentiation, accelerated depletion of the stem cell pool, and the emergence of phenotypically unstable chondrocytes expressing Runx2 and OCN [5,20]. Lgr5 expression declines with OA progression, correlating with loss of Wnt inhibition and a surge in Wnt-activated, osteoblast-like chondrocytes [22]. Conversely, therapeutic Wnt inhibition shows remarkable efficacy: intra-articular sclerostin (SOST) sustains the FCSC pool, restores chondrocyte identity, and repairs cartilage in rabbit and mini-pig models [5,22]. Similarly, SM04690—a Wnt pathway inhibitor—upregulates Wnt16 and anabolic factors (COL2A1, SOX9, and aggrecan) while suppressing catabolic MMP13, thereby promoting FCSC chondrogenesis and preserving the SZ cell pool in vivo [58]. These findings underscore that a Wnt-inhibitory microenvironment is not merely permissive but instructive for FCSC maintenance and proper cartilage homeostasis.

### 4.3. Notch Signaling

Like Wnt signaling, Notch signaling plays a context-dependent, biphasic role in regulating FCSCs within the TMJ condyle, governing their fate specification, differentiation, and contribution to both cartilage homeostasis and TMJOA pathogenesis.

During TMJ morphogenesis, Notch activity is present in the SZ niche where FCSCs reside, as demonstrated by CBF:H2B-Venus reporter mice. Constitutive activation of the Notch1 intracellular domain (NICD) in FCSCs promotes their differentiation toward chondrogenic and osteogenic lineages while suppressing adipogenesis, as evidenced by upregulated *Sox9*, *Acan*, *Col2a1*, *Runx2*, and *Ocn* expression, along with enhanced Alizarin Red and aggrecan staining [59]. Bulk RNA-seq of rabbit TMJ tissues also suggests that Notch signaling is enriched in the SZ relative to underlying condylar cartilage, implicating its probable role in regulating the FCSC fate [60].

Notch signaling is aberrantly activated in TMJOA. In tumor necrosis factor alpha (TNF-α)-induced TMJ arthritis, Notch1, Notch2, Notch4, Jagged1, DLL4, and the downstream targets Hes1/Hes3 are significantly upregulated. Notch1 co-localizes with Col2a1+/Runx2+ cells undergoing cartilage-to-bone differentiation, suggesting that Notch drives pathological FCSC fate switching under inflammatory conditions. DAPT(N-[N-(3,5-difluorophenacetyl)-L-alanyl]-S-phenylglycine t-butyl ester)-mediated Notch inhibition reduced *Notch1*, *Runx2*, and *Ocn* expression in osteogenically cultured FCSCs, indicating that suppressing Notch restrains FCSC osteogenic differentiation and may block pathologically cartilage-to-bone transformation in TMJ arthritis [59]. Similarly, in discectomy-induced TMJOA, Notch1, Jagged1, and Hes5 are significantly upregulated in a time-dependent manner, with expression extending from the superficial layer into deeper zones as degeneration progresses, while Hes1 is initially suppressed but rebounds at later stages [61]. Pharmacological inhibition of Notch signaling with DAPT partially rescues the elevated expression of these molecules and significantly delays cartilage degradation, though it does not fully arrest disease progression [62].

In summary, physiological, transient Notch signaling maintains FCSC chondrogenic potential and cartilage homeostasis, while sustained, pathological Notch activation drives FCSCs toward osteogenic differentiation, promotes chondrocyte differentiation into osteoblasts, and induces inflammatory and catabolic cascades—ultimately depleting the FCSC pool and accelerating OA progression (Figure 2). Thus, Notch inhibition represents a potential therapeutic avenue for TMJOA by preserving FCSC identity and blocking pathological cartilage-to-bone transformation.

### 4.4. Hedgehog Signaling

Hh signaling, primarily mediated by Indian hedgehog (Ihh), is a central regulator of the progenitor population within the mandibular condylar cartilage. Ihh is produced by prehypertrophic chondrocytes and diffuses to the apical progenitor zone, where its receptors (Ptc1, Smo) and effectors (Gli1, Gli2, Gli3) are expressed [4,63]. Although the early literature does not uniformly employ the term “FCSC”, the cell populations regulated by Hh signaling—polymorphic/progenitor layer cells and SZ cells—are spatially and functionally consistent with the FCSCs later defined by Embree and colleagues [4,63,64,65].

During development, Ihh^−/−^ and Gli2^−/−^ embryos exhibit severe condylar hypoplasia, reduced progenitor proliferation and Sox9 expression, and complete absence of the articular disc. Conditional *Smo* ablation in Sox9+ progenitors permits disc formation but prevents disc–condyle separation, establishing two distinct Hh-dependent steps: disc induction (Ihh/Gli2-dependent) and disc–condyle separation (Smo-dependent) [4,63]. Postnatally, Ihh maintains the FCSC pool and its chondrogenic capacity. *Ihh* ablation (Col2a1-CreER or Aggrecan-CreER) causes thinning of the polymorphic/progenitor layer, reduced Ki67+ proliferation, increased apoptosis, decreased Sox9 and fibrocartilage matrix expression, and progressive subchondral bone deterioration [64,65]. EdU tracing confirmed that tenascin C (Tnc)+/Sox9+ progenitors continuously generate chondrocytes, a process disrupted by *Ihh* loss. In vitro, SHH treatment promotes chondrogenesis and alkaline phosphatase activity in condylar apical cells, whereas HhAntag inhibits these effects [65]. Thus, Hh signaling drives FCSC proliferation and chondrogenic differentiation, sustains condylar growth and homeostasis. However, in a collagenase-induced rabbit TMJOA model, NEL-like molecule-1 (Nell-1) was downregulated, whereas Ihh and Shh were aberrantly elevated in superficial-zone FCSCs; restoring Nell-1 suppressed Hh signaling, increased Sox9, Col2a1 and Aggrecan expression, preserved the FCSC pool, and attenuated cartilage degradation and subchondral bone remodeling [66]. In summary, Hh signaling is indispensable for condylar and disc development and postnatal FCSC maintenance but is aberrantly overactivated in osteoarthritis, where dampening Hh activity is associated with preservation of the FCSC pool and reduced cartilage and subchondral bone destruction (Figure 2).

In TMJOA, aberrant mechanical loading activates Hh signaling in Gli1+ osteogenic progenitors, driving their excessive expansion and uneven osteogenic differentiation, which leads to abnormal subchondral bone remodeling and cartilage degeneration. Selective inhibition of Hh signaling in Gli1+ cells with GANT61, or specific genetic inhibition of Hh signaling (*Gli1-Cre^ERT2^*; *Smo^fl^*^/*fl*^) reverses the severe phenotype of TMJOA [31]. Furthermore, YAP activation in Gli1+ osteogenic progenitors suppresses Hedgehog-interacting protein (Hhip) expression, thereby derepressing Hh-Gli1 signaling and promoting excessive osteogenesis that drives aberrant subchondral bone remodeling in TMJOA. Targeting the YAP–Hhip axis in these progenitors attenuates TMJOA progression [34].

### 4.5. PTH1R Signaling

PTHrP (parathyroid hormone-related protein) and PTH (parathyroid hormone) exert their biological effects through binding to PTH1R, a G protein-coupled receptor that transduces downstream signaling cascades upon ligand engagement. PTHrP and PTH act through distinct but interconnected signaling axes to control FCSC proliferation, chondrogenic differentiation, and cartilage homeostasis.

During embryonic development, PTHrP is expressed by SZ cells and diffuses across the condylar cartilage to establish a concentration gradient [4,67]. This gradient acts on PTH1R-expressing cells in the polymorphic layer, where it maintains Runx2 expression to promote chondrocyte precursor proliferation while preventing premature hypertrophic differentiation [67]. In the absence of PTHrP, condylar morphogenesis is severely disrupted, with reduced cell proliferation, diminished Sox9+ chondrocyte domains, and premature chondrocyte exhaustion [4,67].

In the bilateral anterior elevation (BAE) model—in which the occlusal vertical dimension is raised by prosthetic metal tubes bonded to the incisors of 6-week-old mice, with condyles analysed 3, 7 and 11 weeks after induction—mechanical stimulation upregulates the calcium-sensing receptor (CaSR) in Prg4+ SZ cells; in vitro, fluid flow shear stress was applied to superficial-zone cells at 16 dyn/cm^2^ (1.6 Pa) for 2–4 h; CaSR acts upstream to activate the PTHrP nuclear localization sequence (NLS; residues 87–139), promoting proliferation and early differentiation. *CaSR* ablation suppresses PTHrP expression and cartilage thickening, while PTHrP87–139 peptide rescues these defects, confirming PTHrP-NLS as the downstream effector [68]. Xu et al. further established that negative pressure—applied in vitro at −10 kPa for 4 h with a negative-pressure pump (XBL-01LBP; AIPU, China), and induced in vivo by BAE using metal tubes 1.5 mm and 3.0 mm in length on the maxillary and mandibular incisors, respectively, in 6-week-old mice for 7 weeks—activates tuberous sclerosis complex 1 (TSC1)–mechanistic target of rapamycin (mTOR) signaling in CD90+ chondrocytes, which upregulates PTHrP expression. mTOR ablation blocks BAE-induced proliferation, whereas TSC1 deletion enhances it. The effect is strictly PTHrP-NLS-dependent, as NLS mutation abolishes the response and exogenous PTHrP87–139 peptide restores it [69]. Extending these findings, the same group showed—in 6-week-old or 1-year-old female C57BL/6J mice subjected to BAE for 7 weeks (*n* = 6)—that CD90+ chondrocytes exert reparative effects that depend on dual PTHrP–PTH1R signaling. PTHrP-NLS drives proliferation, while PTH1R signaling delays terminal differentiation. Repeated transplantation of CD90+ chondrocytes rescued osteoarthritic cartilage loss, and these effects could be diminished by Pth1r mutation but preserved after PTHrP-NLS knockout [70]. Of note, although these studies have not employed in vivo lineage tracing to definitively establish whether CD90+ or Prg4+ cells constitute FCSC subpopulations, their consistent localization within the superficial fibrocartilaginous layer—the recognized stem cell niche—strongly supports the notion that PTH1R signaling is critically involved in FCSC fate decisions.

In partial discectomy-induced TMJOA models, exogenous PTH (1-34) treatment increases Ctsk+ FCSC proliferation and Col2a1+ chondrogenic differentiation. However, these effects are markedly diminished upon FCSC ablation using *Ctsk-Cre*; *DTR* (*diphtheria toxin receptor*) mice, confirming that PTH’s therapeutic efficacy is mediated through FCSCs [23]. Collectively, these findings establish PTHrP/PTH signaling as a central regulator of FCSC fate across development, homeostasis, and disease, highlighting the PTHrP/PTH and PTH1R axes as promising therapeutic targets for TMJOA.

### 4.6. Other Signaling

Beyond the pathways discussed above, several additional signaling cascades regulate FCSC behavior, although they have received comparatively less attention in the TMJ literature [71,72,73]. The inflammatory TNFα/nuclear factor kappa-B (NF-κB) axis is an important regulator of FCSC fate in TMJOA, a role supported by direct FCSC-specific evidence. TNF-α and interleukin-1β (IL-1β) are elevated in osteoarthritic synovial fluid and drive matrix degeneration [74,75]. In the TMJ, the TNF-α receptor Tnfrsf1b is highly expressed in the FCSC-harboring superficial-zone niche, and TNF-α regulates FCSC stemness in a dose-dependent manner: excessive TNF-α increases apoptosis, suppresses proliferation, and impairs osteogenic and chondrogenic differentiation; these defects are partially reversed by etanercept in vitro. Lineage tracing in Gli1-CreER+; Tm^fl/fl^ mice showed that the chondrogenic output of Gli1+ FCSCs is suppressed in osteoarthritic cartilage and restored by systemic etanercept, indicating that excessive TNF-α functionally exhausts the FCSC pool in vivo. NF-κB is the principal downstream effector: its blockade with BAY 11-7082 preserved FCSC function in rats with TMJOA, whereas intra-articular etanercept alleviated cartilage degeneration and condylar growth retardation in rabbits with TMJ anterior disc displacement [71].

The insulin-like growth factor (IGF) axis, the classic anabolic pathway of cartilage [76], also has a genetically defined FCSC-specific role. Comparison of conditional *Igf1* deletion in Gli1+ FCSCs and in Col2+ chondrocytes revealed temporally distinct functions: chondrocyte-derived *Igf1* sustains early matrix secretion, whereas FCSC-derived *Igf1* maintains long-term cell number, zonation, and matrix output [72]. FCSC-specific *Igf1* deletion abolished phospho-AKT signaling across all cartilage layers and aggravated osteoarthritis in the unilateral anterior crossbite model (UAC: curved metal tubes 5–8 mm in length bonded to the left mandibular incisor with zinc phosphate cement, analysed 4 weeks after induction), identifying IGF-1 as both a physiological growth regulator and a brake on TMJOA progression [72] (Figure 2). Phosphoinositide 3-kinase (PI3K)/protein kinase B (AKT) signaling acts downstream of IGF1R and is essential for joint homeostasis, but its dysregulation in either direction is pathological [77]. In human condylar osteochondroma, hFCSCs were depleted, apoptosis-prone, and biased toward hypertrophic differentiation-changes driven by overactivated PI3K/AKT signaling and upregulation of the pro-apoptotic factor BCL2-like protein 11 (BIM); the PI3K activator 740Y-P reproduced these defects in mice [73]. Together with the *Igf1*-deletion phenotype [72], these findings indicate that PI3K-AKT activity must be maintained within an optimal window: insufficient signaling deprives FCSCs of survival and proliferative support, whereas excessive signaling drives apoptosis and hypertrophic mis-differentiation. Collectively, these three axes integrate inflammatory, anabolic, and survival inputs, with the net effect of each depending on ligand dosage and disease stage.

### 4.7. Signaling Crosstalk in FCSC Fate Regulation

Although the pathways above are conventionally discussed in isolation, FCSC fate is ultimately determined by their crosstalk. First, mechanotransduction feeds into canonical developmental pathways: Piezo1-mediated Ca^2+^ influx activates YAP/TAZ, which may interact with β-catenin [56,78], and repress Hhip to derepress Hh-Gli1 signaling [34], thereby linking mechanical loading to both Wnt and Hedgehog outputs. Second, the canonical Ihh-PTHrP negative-feedback loop couples progenitor proliferation with chondrocyte hypertrophy, integrating Hedgehog and PTH1R signaling across the fibrocartilage zones [67]. Third, IGF-PI3K-AKT signaling acts as a dosage-sensitive node that maintains FCSC fate within a physiological range, while its loss or hyperactivation leads to FCSC depletion and pathological differentiation [72,73]. Finally, Wnt and Notch regulate each other in a reciprocal, context-dependent manner, as documented in the intestine and bone marrow [79,80]; whether such crosstalk also operates in the fibrocartilage remains to be determined.

Collectively, restoring the balance of these regulatory cues may reactivate endogenous FCSCs and facilitate cartilage regeneration, representing a potential therapeutic avenue for TMJOA [6,7] (Table 2).

## 5. Discussion

### 5.1. Temporal Dynamics of FCSC Fate Regulation

FCSC regulation is profoundly stage-dependent, and the major developmental pathways govern TMJ formation with a clear temporal division of labor. During embryonic morphogenesis, Hedgehog signaling is the dominant pathway: TMJ formation requires two distinct, sequentially acting Hh-dependent steps—a Gli2-dependent step that establishes the growth-plate-like cellular organization of the condyle and permits disc formation, and a subsequent, chondroprogenitor-autonomous Smo-dependent step that drives disc–condyle separation [63].

Hh–PTHrP coupling likewise displays a pronounced stage-dependent division of labor: it is indispensable for TMJ morphogenesis and condylar growth in the embryo [4,63]. During postnatal growth, the Hh–PTHrP feedback loop shifts toward pacing chondroprogenitor proliferation and the proliferative-to-hypertrophic transition, with a PTHrP gradient driving condylar chondrogenesis via Runx2 [64,65,67]. In adult homeostasis, pathway activity subsides to low tonic levels that sustain superficial-zone maintenance, and it is the pathological re-elevation of this signaling—rather than its physiological tone—that drives osteoarthritic remodeling [31,66,68,69,70].

Wnt/β-catenin signaling follows a stage-dependent trajectory [21,22]. During embryonic and early postnatal development, canonical Wnt activity (Axin2+) is relatively high in the superficial layers, yet spatially partitioned: Wnt-responsive cells occupy the outermost perichondrium, whereas subjacent chondroprogenitors are kept Wnt-inactive by the Lgr5+/DKK3 inhibitory niche, whose ablation obliterates the condylar blastema [22]. Around weaning, the balance shifts: FSP1+ stem/progenitor cells—derived from the Wnt-responsive Axin2+ lineage—emerge postnatally as Axin2+ cells decline, indicating that the postnatal progenitor compartment is established through a Wnt-on-to-Wnt-off transition that must be actively maintained to preserve the FCSC pool [21]. Forced Wnt activation abolishes FSP1 and causes progressive cartilage loss [21]. In adulthood, the Lgr5+ Wnt inhibitory niche persists but declines with aging and osteoarthritis, releasing Wnt-activated, phenotypically unstable chondrocytes [22]. Thus, Wnt signaling is sequentially redeployed rather than simply switched on or off: spatially restricted during morphogenesis, actively suppressed once the postnatal FCSC compartment forms, and pathologically reactivated in aging and TMJOA [21,22]. Similarly, Notch and Piezo1 exert biphasic, context-dependent functions: they confer protective effects at physiological levels, but become pathological upon overactivation or sustained activation [31,59].

A parallel temporal division exists at the population level. Lgr5+ cells are enriched in the SZ of juvenile condylar cartilage but decline with aging and in osteoarthritis; FSP1+ cells emerge only after birth, expanding from a few superficial cells at P1 to a robust superficial-zone population by P28. These staggered onsets indicate that the FCSC compartment is assembled sequentially during postnatal development rather than being laid down as a fixed embryonic pool. In TMJOA models, FCSC responses follow a stereotyped sequence: an early compensatory proliferative burst, an intermediate phase of aberrant differentiation, and a late phase of pool exhaustion and niche collapse. Therapeutically, this implies that interventions must be stage-matched: anabolic FCSC reactivation may be beneficial early but futile (or even deleterious) once the pool is exhausted, whereas late-stage disease may require combined regenerative and anti-inflammatory strategies.

### 5.2. The FCSC Niche in Homeostasis and Osteoarthritis

FCSC behavior is governed not only by cell-intrinsic pathways but also by a multi-layered niche comprising the extracellular matrix, neighboring mesenchymal (e.g., Lgr5+ niche cells) and skeletal cells (e.g., osteocytes), vascular and neural elements, and immune cells. The extracellular matrix of SZ, provides both biochemical and biophysical cues: a type I collagen-rich fibrous matrix with lubricin/Prg4 at the articular surface [81], matrix-bound latent growth factors [82], and a stiffness profile that directly modulates YAP/TAZ activity [83]. MMP13- and ADAMTS5-mediated degradation of this matrix in TMJOA alters stiffness, releases bioactive matrix fragments, removes quiescence-maintaining signals, and may contribute to FCSC activation and exhaustion [84,85,86].

The mesenchymal and skeletal components of the niche couple FCSC activity to cartilage growth and bone remodeling. Underlying proliferative and hypertrophic chondrocytes provide the Ihh source and PTHrP responsiveness that link FCSC output to condylar growth [4,64,65,67]; Lgr5+ secretory cells create a Wnt-inhibitory microenvironment that preserves FCSC quiescence [22]; the subchondral bone compartment, through Dmp1+ osteocyte signaling and remodeling activity, feeds back onto the overlying fibrocartilage, as demonstrated by the cartilage degeneration secondary to skeletal hypophosphatemia in Dmp1-null mice [19]. Although condylar cartilage has long been considered avascular based on rodent data, studies in miniature pigs and humans have revealed that all layers of the condylar fibrocartilage contain CD31+/α-SMA+ vessels during growth. Osteoarthritic remodeling further increases vascular density—accompanied by elevated vascular endothelial growth factor A (VEGFA) secretion from diseased FCSCs themselves—while vascular invasion and sensory nerve sprouting additionally reshape the osteoarthritic niche [87].

Immune cells are integral components of the FCSC niche rather than passive onlookers, and in TMJOA they become dominant niche occupants. Synovial macrophages, polarized toward a pro-inflammatory phenotype, together with synovial fibroblasts, release TNF-α, IL-1β, IL-6, and extracellular vesicles that may act on FCSCs to suppress chondrogenesis and accelerate pool exhaustion [59,71,88]; T cells contribute IL-17A, which synergizes with TNF-α to amplify matrix degradation. An immunometabolic axis links pathological Piezo1 activation in subchondral mesenchymal cells to Ca2+-STAT1-HK2 glycolytic reprogramming and MIF secretion, recruiting Th17 cells that drive osteoclast-mediated bone erosion and secondary cartilage destruction. Mesenchymal stromal cell–mast cell interplay has likewise been implicated in musculoskeletal tissue repair and may represent an additional, as-yet-unexplored axis of the TMJ immune niche [89].

Conversely, protective immune–niche circuits also exist. Eosinophils, which are more abundant in the synovial fluid of healthy individuals than in that of patients with TMJOA, stimulate synovial fibroblasts to secrete lumican. Lumican then binds Annexin A1 on chondrocytes and suppresses TGF-β2/SMAD2/3 signaling, thereby alleviating synovitis, cartilage degradation, and subchondral bone erosion [90]. Notably, despite well-documented synovitis in TMJOA, direct evidence of synovial-FCSC communication is still lacking: no study has yet exposed FCSCs to TMJOA synovial fluid or synovium-derived extracellular vesicles. To date, the dose-dependent regulation of FCSC stemness by synovial-fluid-borne TNF-α remains the clearest indirect evidence [71].

### 5.3. FCSC Exhaustion: Primary Driver or Secondary Consequence of TMJOA?

Whether FCSC exhaustion is a primary cause of TMJOA or a downstream consequence of chronic mechanical stress, inflammation, and matrix degradation remains a central question. To date, the available evidence supports both positions.

In favor of a primary role, genetic ablation of FCSCs (*FSP1-Cre*; *DTA* or *Gli1-Cre^ERT2^*; *DTA*) alone reproduces osteoarthritic degeneration [20,21]; and therapies that restore FCSC function prevent or reverse degeneration [23]. For example, PTH promotes the proliferation and chondrogenic differentiation of FCSCs in vivo and alleviates TMJOA, indicating that FCSC status is causally linked to tissue integrity [23]. In favor of a secondary role, aberrant mechanical loading (through excessive Piezo1 activation) [33], inflammatory cytokines (through NF-κB or Notch) [59,71], and niche destruction (through matrix degradation or loss of Wnt-inhibitory niche cells) [22,81,84] each independently deplete the FCSC pool, indicating that exhaustion can be driven by upstream insults.

The most plausible interpretation is a feed-forward model. Upstream triggers (mechanical, inflammatory, or both) initiate FCSC dysfunction. FCSC exhaustion amplifies and perpetuates disease by abolishing the tissue’s intrinsic repair capacity. This in turn promotes further matrix loss, inflammatory amplification, and niche collapse. This model has direct therapeutic implications: early-stage disease should be managed by removing the trigger and protecting the FCSC pool, whereas late-stage disease—once the pool is exhausted and the niche destroyed—will require combined regenerative strategies. Longitudinal FCSC fate mapping from health through early to late TMJOA will be required to define the point at which exhaustion becomes irreversible.

### 5.4. Evidence from Human TMJ Tissues

Most of the mechanistic evidence discussed above derives from genetically engineered mouse models, and its translation to humans requires caution and validation. Human data now span development, homeostasis, and disease. Single-cell profiling of human embryonic condyles at 3 and 4 months of gestation (7052 and 9572 cells, respectively; the number of embryos at each stage was not specified) revealed a multilineage progenitor hierarchy: MSCs pass through a transition state to generate osteoblasts, hypertrophic chondrocytes, and tenocytes [91]. These findings establish that the progenitor heterogeneity described in the murine FCSC model is hard-wired during human development.

In adult human condyles, hFCSCs were prospectively isolated from the SZ, where they displayed a marked chondrogenic capacity that requires SOX9 [17]. Human disease studies independently corroborate the murine mechanisms. In patients with TMJOA, SOX9 is selectively suppressed in the superficial zone, providing the first clinical evidence that the FCSC chondrogenic program is extinguished in disease [17]. In condylar osteochondroma, hFCSCs exhibit pool depletion and hypertrophic-biased chondrogenesis driven by overactivated PI3K/AKT signaling [73]. In aging and TMJOA, human condylar chondrocytes lose their identity and the Lgr5+ Wnt-inhibitory niche collapses; moreover, the Wnt inhibitor StemJEL restores chondrocyte identity in primary human osteoarthritic cells [22]. PIEZO1 is the most highly expressed mechanosensitive ion channel in human condylar cartilage and bone [33]. Osteoarthritic human TMJ tissues exhibit increased vascular density, together with elevated VEGFA secretion from diseased FCSCs themselves [87]. Single-cell analysis of human TMJOA cartilage further uncovered neurovascular–osteochondral crosstalk, with a resident progenitor population sitting at the root of this differentiation trajectory; this pilot dataset comprised only two donors (one osteoarthritic condyle and one condyle with benign condylar hyperplasia as a control) [92].

Translation nonetheless faces caveats. Human condylar fibrocartilage is thicker and more highly stratified than murine tissue. Genetic lineage tracing cannot be performed in human samples, and most TMJOA specimens are obtained from patients with end-stage disease. Accordingly, validated human FCSC markers remain lacking. Moreover, several available human condylar single-cell atlases are based on only one or two donors, which limits assessment of interindividual variation. We consider building single-cell and spatial atlases of healthy and osteoarthritic human condyles to be the field’s highest-priority next step.

### 5.5. Strength of Evidence and Statistical Limitations

The strength of evidence also varies considerably across the field, and conclusions should be weighted accordingly. Causal claims rest primarily on in vivo genetic lineage tracing and ablation studies, whereas transcriptomic inference—such as the proposed Crabp1+ progenitor population—remains correlative until validated by lineage tracing. Moreover, most lineage-tracing studies in this field use three to six animals per group. As estimated by power analysis based on the noncentral t distribution (two-tailed α = 0.05, power = 0.80), such samples can detect only very large effects (minimum detectable standardized difference d ≈ 3.07 for *n* = 3, 2.02 for *n* = 5, and 1.80 for *n* = 6), whereas detecting a moderate effect (d = 1.00) would require 17 animals per group. Quantitative findings from these studies should therefore be interpreted with attention to effect sizes and variability rather than to nominal percentages alone [93]. The same caution applies to the human transcriptomic evidence: the human condylar single-cell datasets available to date comprise one donor [33] or two donors [92], so donor-level variation cannot be estimated and these atlases should be treated as hypothesis-generating until replicated across independent specimens.

These considerations frame the limitations and future directions discussed in Section 6, in which interspecies differences, spatial resolution, mechano-chemical coupling, and delivery barriers are addressed in turn.

## 6. Limitations and Future Perspectives

Although substantial progress has been made in the research on FCSCs and the regulatory mechanisms governing their cell fate, important limitations remain on the path toward the definitive cure for TMJOA and the achievement of functional cartilage regeneration; these limitations also point to directions for future investigation.

### 6.1. Interspecies Differences and Refinement of Human Single-Cell Atlases

Nearly all current lineage-tracing studies of Ctsk+, Gli1+, and Scx+ populations rely on transgenic mouse models based on the Cre-loxP system. Nevertheless, objective differences exist between humans and rodents in TMJ anatomical dimensions, biomechanical transduction patterns (e.g., mastication modes), and the specificity of stem cell markers [94]. It remains unclear whether murine FCSC markers are conserved and biologically relevant in human condylar tissues. Future studies will require larger human TMJ tissue biobanks covering both healthy and pathological conditions. A comprehensive atlas of human TMJ stem cell heterogeneity is needed to determine whether findings obtained from mouse models can be faithfully translated to humans.

### 6.2. Lack of Dynamic Three-Dimensional Spatial Resolution (Spatial Transcriptomics)

Stem cell fate is strongly governed by the niche. Conventional flow cytometry and single-cell RNA sequencing lose the three-dimensional spatial coordinates of cells during tissue dissociation. Spatial transcriptomics should therefore be incorporated into future studies [95,96]. By dissecting cell–cell interaction networks within the SZ, PZ, CZ, HZ, and subchondral bone, we may elucidate how FCSCs communicate and interact with niche cells.

### 6.3. Precise Simulation of Mechano-Chemical Coupling and Microfluidic Technology

The in vivo mechanical milieu (tensile stress, compressive stress, shear force) is dynamic and composite, whereas current in vitro cell loading models (e.g., uniaxial stretching, static compression) are often oversimplified. The magnitude, duration, and frequency of mechanical loading that distinguish physiological FCSC activation from pathological exhaustion remain unclear. Future research should leverage organoid technology and joint-on-a-chip platforms to engineer miniature TMJ systems in vitro that incorporate vascular networks, immune cells (e.g., macrophages), and multilineage stem cells [97,98]. Combined with microfluidic systems to deliver complex biomimetic mechanical loading, these models will more faithfully recapitulate mechano-chemical coupling mechanisms.

### 6.4. Barriers to Clinical Translation of Targeted Delivery Systems

Although agents targeting specific stem/progenitor cells (e.g., PTH, Piezo1 inhibitors, and Wnt modulators) exhibit promising therapeutic effects, all studies remain at the preclinical, animal-model stage, with no TMJ-specific clinical trials to date. Meanwhile, the biosafety of pharmacological interventions remains to be addressed, including the oncogenic risk of systemic Wnt inhibition and potential adverse effects of Piezo1 blockade in mechanosensitive tissues. The translational barriers faced by stem-cell strategies in another fibrocartilaginous tissue—the intervertebral disc—offer an instructive parallel [99].

Breakthroughs in future therapies hinge on developing smart biomaterials with high tissue affinity and sustained-release properties, such as nanoliposomes targeting specific cell membrane receptors and matrix metalloproteinase (MMP)-cleavable responsive hydrogels [100,101]. Spatiotemporally controlled release systems would allow precise delivery of fate-regulating therapeutics to the SZ or subchondral bone, thereby enabling clinically meaningful targeted therapy of endogenous stem cells. Beyond biological and technical barriers, clinical translation also faces substantial regulatory hurdles. No disease-modifying osteoarthritis drugs have been approved for TMJOA, and the field still lacks standardized clinical endpoints and validated outcome measures, which collectively complicate trial design and regulatory review.

## 7. Conclusions

Overall, our understanding of FCSCs has gradually deepened since the concept was first proposed. FCSCs exhibit heterogeneity, and comprise multiple marker-defined populations with distinct anatomical localizations, lineage outputs, and regulatory dependencies; FCSC quiescence is actively maintained by niche-derived Wnt inhibition and physiological Hedgehog-PTHrP signaling, and its disruption is sufficient to initiate osteoarthritic degeneration; FCSC exhaustion and niche collapse form a feed-forward loop with matrix degradation and inflammation, implying that early-stage disease should be managed by removing the upstream trigger and protecting the stem cell pool, whereas late-stage disease will require combined regenerative strategies.

Nevertheless, multiple unresolved issues warrant further investigation. The relative contribution of each subpopulation, and whether these functions change across developmental and pathological stages, remain unclear. In addition, almost all available data derive from in vivo lineage tracing in mice; whether these findings apply to humans requires further verification. Establishing TMJ organ-on-a-chip or TMJ organoid models to characterize specific cell subpopulations and perform targeted drug screening represents a promising avenue for future work.

## Figures and Tables

**Table 1:** Lineage-tracing studies of temporomandibular joint fibrocartilage stem/progenitor cells: transgenic models, induction regimens, lineage output and principal roles.

Cell Population (Marker)	Transgenic Model	Induction Regimen (Stage/Age; Sex; Tamoxifen; Chase)	Lineage Output	Principal Role
αSMA (Acta2)+ [5]	*αSMACre^ERT2^*/*Ai9*;	Tamoxifen 62.5 µg/g, single injection at P16; male; chase 2 d and 15 d. (Rats: EdU 50 µg/g at E16.5–E18.5, chased to newborn, 8 w and 16 w.)	αSMA+ superficial-zone cells give rise to Col2a1+ chondrocytes; descendants increase in the SZ and calcified cartilage from 2 to 15 d. A single FCSC generates a cartilage anlage that remodels into bone and organizes a haematopoietic microenvironment.	First identification of the FCSC niche in the SZ; Wnt activation depletes the FCSC reservoir and causes cartilage degeneration, whereas the Wnt inhibitor sclerostin preserves it.
Scx+ [19]	*Scx-Cre^ERT2^*; *tdTomato*; *Scx-Cre^ERT2^*; *tdTomato*; *DTA*; crossed to *Dmp1*-null. Comparators: *Acan-Cre^ERT2^* and *Col10a1-Cre*; *tdTomato*	Tamoxifen 75 mg/kg i.p. (10 mg/mL in 10% ethanol/90% corn oil); single injection at P3 (harvest P4, P9, P33, P60) or twice weekly for late recruitment; DTA ablation P3→P33. Sex NR.	Scx-lineage cells form the TMJ disc and a new subset of Sox9+ chondrocytes in the condyle (~50% of disc cells and ~18% of condylar cartilage cells by P60); low proliferation (6.6% EdU+ at P19). They do not undergo osteogenesis, unlike Scx− chondrocytes.	Drive postnatal TMJ growth (disc and condyle); the Scx+ chondrocyte subset remains cartilaginous throughout the ages examined (up to 8 months) and is sharply increased by hypophosphataemia in Dmp1-null mice.
Gli1+ [20,30,31]	[30] *Gli1-Cre^ERT2^*; *tdTomato*; *Gli1-LacZ* [31] *Gli1-Cre^ERT2^*; *tdTomato*; *Gli1-Cre^ERT2^*; *Smo^fl^*^/*fl*^; [20] *Gli1-Cre^ERT2^*; *tdTomato*; *Gli1-Cre^ERT2^*; *DTA*; *Gli1-Cre^ERT2^*; *β-catenin^fl^*^/*fl*^	[30] Tamoxifen 1.5 mg/10 g (~150 mg/kg) i.p. at PN0.5, 4 w or 1 mo; chase 3 d–3 mo; fracture model induced at 1 mo; male and female. [31] Induction at 1 mo, chase to 12 mo; unilateral partial discectomy in 6 wk male mice, harvest at 2 and 4 w; GANT61 50 µg/g s.c. every other day; tamoxifen dose NR. [20] Tamoxifen i.p. once daily for 2 d (dose NR) at PN3.5, traced to 12 w; DTA ablation induced at PN 2 w; β-catenin cKO induced at PN3.5 or 4 w. Sex NR.	[30] Gli1+ cells beneath the cartilage give rise to nearly all Osx+ osteoblasts of the subchondral bone; Gli1+ cells in the upper cartilage layers persist; during fracture repair they also contribute to cartilage. [31] Derivatives label the trabecular bone surface (Osx+) throughout 12 mo. [20] About 61.27% ± 6.94% of Gli1+ descendants remain within the SZ throughout 12 w; they give rise to Col II+ chondrocytes and Col X+ hypertrophic chondrocytes.	[30] Gli1+ osteogenic progenitors contribute to postnatal condylar development and fracture repair. [31] Aberrant loading expands Gli1+ osteogenic progenitors and drives TMJOA; blocking Hh signalling (*Smo* deletion or GANT61) alleviates OA. [20] β-catenin is required for Gli1+ FCSC hypertrophic differentiation and their ablation thins the cartilage with SZ cell loss.
Ctsk+ [23,41]	[23] *Ctsk-Cre*; *tdTomato*; *Ctsk-Cre*; *DTR* + diphtheria toxin; constitutive Cre, no tamoxifen [41] *Ctsk-Cre*; *tdTomato*; constitutive Cre, no tamoxifen	[23] Mice: partial discectomy with or without PTH for 4 w; SD rats: 6 wk male, discectomy-induced OA ± PTH; FCSCs isolated from 5 wk rat condyles; males only. [41] MA: 5 wk male mice, lower incisors ground by 1 mm every 3 d for 2 w (rats: 5 wk male SD, 3 mm every 3 d for 2 w); no induction drug.	[23] Ctsk+ cells are confined to the superficial layer at 3 d after birth and differentiated into Col2a1+ chondrocytes in 28-day-old mice. [41] Ctsk+ cells sit in the SZ at 3 d of age; by 2 w they infiltrate the deeper cartilage and differentiate into Col2a1+ mature chondrocytes; MA further increases this output.	[23] PTH alleviates TMJOA by promoting Ctsk+ FCSC proliferation and chondrogenic differentiation; ablation of Ctsk+ cells abolishes the therapeutic effect of PTH. [41] Ctsk+ FCSC s transduce tensile stress into chondrogenic differentiation and cartilage remodeling.
Lgr5+ [22]	*Lgr5^EGFPcre+^*^/*−*^; *Rosa/tdTomato*; *Lgr5^DTR-EGFP^* + diphtheria toxin; *TOPGAL* Wnt reporter; *Lgr5* in situ hybridisation	Tamoxifen 25 mg/mL, daily at E12.5+E13.5 (harvest E19.5, P21, 1 y), E15.5+E16.5 (harvest E19.5), or P1-P3 (harvest P180); DT 50 µg/kg i.p. at E13.5, E15.5, E17.5, analysed 24 h later. Equal numbers of males and females.	Lgr5 progeny populates the disc, the articular cartilage SZ/polymorphic zone and the perichondrium/ramus periosteum; they self-renew during injury but do not differentiate into Col2a1+ chondrocytes.	Secretory niche cells that build a Wnt-inhibitory niche and preserve chondroprogenitor identity; essential early (ablation at E13.5/E15.5 disrupts the condylar blastema), whereas in adults they act mainly as niche support.
FSP1/S100A4+ [21]	*FSP1-Cre*; *mTmG*; *FSP1-Cre*; *DTA*; *FSP1-Cre*; *βcatGOF*. Comparators: *Axin2-Cre^ERT2^*; *tdTomato*; *Mesp1-Cre*; *tdTomato*; BrdU labelling	Constitutive Cre—no tamoxifen. FSP1 is absent at E17.5; DTA ablation produces severe OA (OARSI grade 6) at 16 w. Sex NR.	FSP1+ cells appear as scattered superficial cells at P1, becomes strong by P28, and its clones extend through the hypertrophic and ossification zones by P56–63; and a subset is BrdU label-retaining (slow-cycling).	A postnatally emergent SZ stem/progenitor population essential for condylar growth and homeostasis; it requires a Wnt-off state (constitutive β-catenin activation down-regulates FSP1 and causes progressive cartilage loss).
Crabp1+ [24]	None available; single-cell RNA sequencing only; Crabp1+ In situ hybridization	No induction, no chase; transcriptomic analysis	Not determined by lineage tracing. Crabp1+ inner perichondrial/PZ cells express Nrg1, Lsamp, Tac1 and Igfbp5, consistent with an early chondrogenic transcriptional state.	Atlas-level, hypothesis-generating: a putative early chondrogenic progenitor within the inner perichondrium. Correlative only—awaits lineage-tracing validation.

Reference numbers correspond to the reference list of this manuscript. NR = not reported in the main text; SZ = superficial zone; PZ = polymorphic zone; FCSC = fibrocartilage stem cell; MA = mandibular advancement; DT = diphtheria toxin; DTA = diphtheria toxin fragment A; DTR = diphtheria toxin receptor; GOF = gain-of-function; cKO = conditional knockout; PN = postnatal; i.p. = intraperitoneal; s.c. = subcutaneous; EdU = 5-ethynyl-2′-deoxyuridine; BrdU = 5-bromo-2′-deoxyuridine; OARSI = Osteoarthritis Research Society International; TMJOA = temporomandibular joint osteoarthritis; Ai9 = Rosa26-CAG-LSL-tdTomato Cre reporter; mTmG = membrane-tdTomato/membrane-EGFP Cre reporter; EGFP = enhanced green fluorescent protein; TOPGAL = TCF/LEF–LacZ Wnt reporter; βcat = β-catenin.

**Table 2:** Potential FCSC-directed therapeutic approaches for TMJOA: targeted cell populations, TMJOA models, interventions, mechanisms of action and limitations.

Signaling Pathway	Targeted Cell Population	TMJOA Model (Induction/Species/n/Sex/Age)	Intervention	Mechanism of Action	Limitation
Wnt/β-catenin [5,20,21,22,58]	[5] αSMA+ cells [21] FSP1/S100A4+ stem/progenitor cells [22] Lgr5+ cells [20] Gli1+ FCSCs [58] SZ FCSCs	[5] Disc perforation: NZW rabbits, male 12 wk, *n* = 8; [21] None [22] Disc perforation: minipigs *n* = 44; rabbits *n* = 36; [20] UPD: mice 4 wk, *n* = 3–6/group, sex NR; [58] Partial discectomy: female NZW rabbits 12 wk and female SD rats 6 wk, *n* = 8/group	[5] SOST 100 ng/mL intra-articular injection [21] FSP1-Cre; DTA; FSP1-Cre; β-cat GOF [22] StemJEL intra-articular injection; Lgr5-DTR [20] Gli1-Cre^ERT2^; β-catenin^fl/fl^ [58] SM04690 intra-articular injection	Excessive Wnt/β-catenin signalling drives FCSCs toward hypertrophy and exhausts the SZ pool [5,20]; Lgr5+ cells secrete DKK3 to build a Wnt-inhibitory niche that preserves chondrocyte identity [22]; the FSP1+ pool requires low Wnt activity [21]; Wnt inhibition preserves FCSCs and restores cartilage [5,20,22,58].	[5] Biomechanics and bone phenotype not evaluated; [21] ablation also affects superficial synovial cells; [22] pig/human cells unsorted; [20] Gli1+ and LRCs overlap only ~20–25%; [58] Wnt16 mechanism not validated genetically
Piezo1 [33,41]	[33] Gli1+ FCSCs [41] Ctsk+ FCSCs	[33] UPD: C57BL/6 mice, male 6 wk, *n* = 6/group; [41] MA: Ctsk-Cre mice, 5 wk, *n* = 5–6/group; in vitro 10% equibiaxial strain, 0.5 Hz	[33] Gli1-Cre^ERT2^; Piezo1^fl/fl^; [41] GsMTx4 and BAPTA in vitro; siPrkca intracapsular	[33] Piezo1 mediates overload-induced Gli1+ FCSC pool exhaustion, so its deletion is protective under overloading; [41] Tensile stress → Piezo1 → Ca2+ → Prkca drives FCSC chondrogenesis	[33] Gli1-Cre not FCSC-specific (also active in subchondral bone) [41] Ctsk+ cells heterogeneous (disc and bone marrow)
Notch [59,61,62]	[59] SZ FCSCs [62] SZ chondrocytes	[59] rhTNF-α injection: C57BL/6J mice, both sexes, *n* = 6; [62] Total discectomy: female 8 wk, *n* = 16/group	[59] Lentiviral NICD; DAPT in vitro; [62] DAPT intra-articular injection	Notch activation drives FCSCs toward chondrogenic and osteogenic lineages [59]; DAPT postpones TMJOA degradation [62].	[59] DAPT tested only in vitro; [62] Effect postponed, not inhibited
Hedgehog [31,34,66]	[31,34] Gli1+ osteogenic progenitors [66] SZ FCSCs and mandibular condylar chondrocytes	[31] UPD: mice, male 6 wk, *n* = 3–5/group [34] Same UPD model: mice 6 wk, both sexes, *n* = 5/group [66] Intra-articular collagenase: female NZW rabbits 9–10 wk, *n* = 5/group	[31] Gli1-Cre^ERT2^; Smo^fl/fl^; GANT61 s.c. [34] Gli1-Cre^ERT2^; Yap^fl/fl^; SAG i.p.; verteporfin [66] AAV9-Nell-1 intra-articular injection; AdNell-1 in vitro; cyclopamine	Abnormal loading activats Hh-Gli1 in subchondral bone and drives disordered remodeling [31]; YAP-TAZ represses the Hhip promoter, releasing the brake on Hh signalling [34]; Nell-1 suppresses Ihh/Shh and maintains the SZ FCSC pool [66].	[31] Males only with small group size (*n* = 3–5); [34] Bone phenotype may be secondary to cartilage deformity; [66] Collagenase model is not gradual like clinical TMJOA
PTHrP/PTH [23,68,69,70]	[68] Prg4+ superficial-zone cells [69,70] CD90+ chondrocytes [23] Ctsk+ FCSCs	[68] BAE bite: mice 6 wk, *n* = 6/group [69] BAE: female C57BL/6J mice 6 wk, *n* = 6/group [70] BAE and UAC: female C57BL/6J mice, 6 wk and 1 yr, *n* = 6 [23] Partial discectomy: male SD rats 6 wk; male Ctsk-Cre mice 8 wk; *n* = 3–4	[68] Prg4-Cre^ERT2^-driven CaSR/PTHrP deletion; PTHrP_87–139_; cinacalcet; [69] CD90-CreER-driven Mtor/Tsc1/PTHrP mutation; rapamycin in vitro; PTHrP_87–139;_ [70] CD90-CreER-driven PTHrP/Pth1r mutation; [23] PTH(1-34) intra-articular injection; Ctsk+ FCSC ablation	Loading induces PTHrP via CaSR or TSC1-mTORC1; its NLS (residues 87–139) drives superficial progenitor proliferation independently of PTH1R, while PTHrP(1-34)/PTH1R prevents terminal differentiation [68,69,70]; CD90-CC transplantation or PTH(1-34) reverses cartilage loss [23,70]; CaSR is upstream of PTHrP-NLS, since cinacalcet fails to rescue PTHrP-CKO [68].	[68] PTHrP-NLS model is early-lethal [69] CD90 not chondrocyte-specific (bone marrow and synovial MSCs); [70] All animals female and young; no healthy controls; [23] PTHrP/Ihh and canonical Wnt crosstalk unresolved
TNF-α/NF-κB [71]	[71] SZ FCSCs	[71] Surgical TMJOA: male mice 7 wk, *n* = 9; partial discectomy: male SD rats, *n* = 12; ADD: male NZW rabbits 10 wk, *n* = 12–13	[71] Etanercept s.c. and intra-articular; BAY 11-7082 s.c.; TNF-α in vitro	TNF-α signals via TNFR2 → NF-κB, suppressing FCSC proliferation, inducing apoptosis and blocking chondrogenesis; etanercept or BAY 11-7082 restores FCSC retention and differentiation in TMJOA.	[71] All in vivo work male although women are more susceptible
IGF1/PI3K-AKT [72]	[72] Gli1+ FCSCs	[72] UAC: male mice 6 wk, *n* = 8–9/group	[72] *Gli1-CreER*; *Igf1^fl/fl^*	IGF-1 sustains FCSC proliferation and chondrogenesis via PI3K/AKT; *Igf1* deletion accelerates OA progression.	[72] Autocrine/paracrine IGF-1 network not resolvable by the animal model alone

Reference numbers correspond to the reference list of this manuscript. UPD = unilateral partial discectomy; UAC = unilateral anterior crossbite; BAE = bilateral anterior elevation bite; ADD = anterior disc displacement; GOF = gain-of-function; MA = mandibular advancement; SZ = superficial zone; FCSC = fibrocartilage stem cell; PTHrP = parathyroid hormone-related protein; PTH = parathyroid hormone; LRC = label-retaining cell; s.c. = subcutaneous; i.p. = intraperitoneal; i.a. = intra-articular; NR = not reported.

## Data Availability

No new data were created or analyzed in this study. Data sharing is not applicable to this article.

## Abbreviations

The following abbreviations are used in this manuscript:

α-SMA	alpha-smooth muscle actin
Acan	aggrecan
Acta2	actin alpha 2, smooth muscle
ADAMTS5	a disintegrin and metalloproteinase with thrombospondin motifs 5
AKT	protein kinase B
AMPK	AMP-activated protein kinase
Axin2	axis inhibition protein 2
BrdU	5-bromo-2′-deoxyuridine
CaSR	calcium-sensing receptor
Col X	collagen type X
Col1	collagen type I
Col2a1	collagen type II alpha 1 chain
CPC	chondroprogenitor cell
Crabp1	cellular retinoic acid-binding protein 1
Ctsk	cathepsin K
DAPT	N-[N-(3,5-difluorophenacetyl)-L-alanyl]-S-phenylglycine t-butyl ester
DKK3	dickkopf WNT signaling pathway inhibitor 3
DLL4	delta-like ligand 4
Dmp1	dentin matrix protein 1
DTA	diphtheria toxin fragment A
EdU	5-ethynyl-2′-deoxyuridine
ERK	extracellular signal-regulated kinase
FCSC	fibrocartilage stem cell
FSP1	fibroblast-specific protein 1
Gli1	glioma-associated oncogene 1
Gli2	GLI family zinc finger 2
GsMTx4	grammostola mechanotoxin 4
hFCSC	human fibrocartilage stem cell
Hhip	hedgehog-interacting protein
HK2	hexokinase 2
Igf1	insulin-like growth factor 1
IGF	insulin-like growth factor
Ihh	Indian hedgehog
IL-1β	interleukin-1 beta
IL-6	interleukin-6
Lgr5	leucine-rich repeat-containing G protein-coupled receptor 5
LRC	label-retaining cell
MIF	macrophage migration inhibitory factor
MMP13	matrix metalloproteinase 13
MSC	mesenchymal stem/stromal cell
mTOR	mechanistic target of rapamycin
NF-κB	nuclear factor kappa B
NLS	nuclear localization sequence
OA	osteoarthritis
OARSI	Osteoarthritis Research Society International
OCN	osteocalcin
Piezo1	piezo-type mechanosensitive ion channel component 1
PI3K	phosphoinositide 3-kinase
Prg4	proteoglycan 4
PTH	parathyroid hormone
PTH1R	parathyroid hormone 1 receptor
PTHrP	parathyroid hormone-related protein
Runx2	runt-related transcription factor 2
S100A4	S100 calcium-binding protein A4
scATAC-seq	single-cell assay for transposase-accessible chromatin with high-throughput sequencing
scRNA-seq	single-cell RNA sequencing
Scx	scleraxis
Smo	smoothened, frizzled class receptor
SOST	sclerostin
Sox9	SRY-box transcription factor 9
STAT1	signal transducer and activator of transcription 1
SZ	superficial zone
TAZ	transcriptional coactivator with PDZ-binding motif
Th17	T helper 17
TMJ	temporomandibular joint
TMJOA	temporomandibular joint osteoarthritis
TNF-α/TNFα	tumor necrosis factor alpha
TRP	transient receptor potential
TSC1	tuberous sclerosis complex 1
VEGF	vascular endothelial growth factor
VEGFA	vascular endothelial growth factor A
YAP	yes-associated protein
